# The Microbiota as a Potential Cause of Disease

**DOI:** 10.3390/diseases14070260

**Published:** 2026-07-20

**Authors:** Giusi Santangelo, Lucrezia Lamorgese, Noemi Tonti, Maria Grazia Porpora, Innocenza Palaia, Federica Tomao, Violante Di Donato, Margherita Fischetti, Daniele Di Mascio, Antonella Giancotti, Giorgia Perniola, Ludovico Muzii

**Affiliations:** Department of Gynecological, Obstetrical and Urological Sciences, “Sapienza” University of Rome, Policlinico Umberto I, Viale Regina Elena, 328, 00161 Rome, Italy

**Keywords:** microbiota, inflammaging, eubiosis, diet, gut diseases, cardiovascular diseases, diabetes, osteoporosis, gut–brain axis, short-chain fatty acids

## Abstract

**Background:** The human microbiota plays a crucial role in maintaining physiological homeostasis and influencing the development of chronic diseases not only in the gut but in the whole body. **Material and Methods:** This literature review is based on a comprehensive search of the PubMed database covering the period from 2008 to 2026. Approximately 65 key studies were included in the final analysis. Only articles published in English were included. The search included keywords such as microbiota, inflammaging, eubiosis, diet, gut diseases, cardiovascular diseases, diabetes, and osteoporosis. This narrative review explores the composition, development, and functional significance of the gut microbiota across the human lifespan, highlighting its dynamic interaction with environmental factors. Early-life microbial colonization, shaped by factors including delivery mode and breastfeeding, has long-term implications for immune system maturation and disease susceptibility. **Results:** A balanced gut microbiota (eubiosis) supports host health through metabolic activities, mainly by the production of short-chain fatty acids (SCFAs), which regulate intestinal barrier integrity, immune responses, and systemic inflammation. Contrarily, dysbiosis—characterized by reduced microbial diversity and an overrepresentation of pro-inflammatory species—is associated with chronic low-grade inflammation (inflammaging) and contributes to the pathogenesis of multiple diseases. Age-related changes in microbial composition are shown to activate inflammatory processes and impair immune regulation, thereby increasing disease risk. Therefore, it is important to recognize the role of microbiota alterations in key pathological conditions, including neurodegenerative diseases, cardiovascular diseases, type 2 diabetes mellitus, and osteoporosis. **Conclusions:** Finally, the potential of microbiome-targeted interventions, such as probiotics, prebiotics, and dietary modulation—in particular the Mediterranean diet is recognized as the most balanced—is discussed as a promising strategy to restore microbial balance and mitigate inflammaging. Further research is needed to better understand the association between microbiota and host health and to optimize therapeutic approaches for aging populations.

## 1. Introduction

The human microbiota is a highly complex and dynamic ecosystem composed of diverse microbial communities, including bacteria, viruses, fungi, archaea, and protozoa, that colonize distinct anatomical niches, while microbiome refers to the collective genomic content of all microorganisms comprising the microbiota, that is, their genetic material and the potential biological functions encoded by their genes [1]. The human body hosts about 10^17^ microorganisms, which is approximately ten times the number of cells that constitute a human being. These microorganisms are distributed in colony-like groups on different sites of the human body; the most well known is the gut, but they can also be found in the oral cavity, skin, airways, and other locations. At each site of the human body these microorganisms, mainly bacteria, share similar characteristics that allow them to cooperate with the host in a way that is beneficial to both [2]. During human evolution the environment underwent critical changes such as those in climate, temperature and diet; all of these affected the microbiota, which evolved under selective pressure [3]. This coexistence is allowed by the host’s immune system, which developed a complex mechanism to recognize and remove invading microbes, while preserving the microorganisms that are a part of the microbiota [4]. The gut microbiota is the most studied and the largest in the human body; it is made of up to 400 species, most of which belong to the Bacteroidetes, Firmicutes, Actinobacteria, and Proteobacteria phyla [5]. Each millimeter of the large intestine hosts about 10^11^ microbial cells [6]; the relative proportion of microorganisms changes between individuals and throughout the course of each person’s life [7].

## 2. Methods

This narrative synthesis was conducted following a comprehensive literature search in the PubMed database spanning the years 2008 to 2026, with the final search performed in May 2026. The search strategy employed a combination of Medical Subject Headings (MeSHs) and free-text keywords including “microbiota,” “inflammaging,” “eubiosis,” “diet,” “gut diseases,” “cardiovascular diseases,” “diabetes,” and “osteoporosis,” aiming to capture a broad spectrum of the relevant literature. The search was limited to articles published in English.

The initial search yielded a substantial number of articles, which were subsequently screened by title and abstract to assess their relevance to the core topics of microbiota composition, underlying mechanisms, and associated clinical implications. Potentially eligible studies underwent full-text review to allow a more detailed assessment of methodologies, findings, and thematic relevance. A total of 65 studies were ultimately included, selected according to predefined inclusion criteria restricting the review to original research articles and review papers published in English and available in full text. Conference abstracts, case reports, editorials, letters, and studies published in languages other than English were excluded to ensure data reliability and consistency. Additionally, the reference lists of pertinent articles were manually examined to identify further studies meeting the inclusion criteria.

Given the conceptual and evolving nature of the microbiota field, along with existing knowledge gaps and variability in study designs, a narrative review format was preferred over a systematic review or meta-analysis. This approach enabled an integrative synthesis of clinical and mechanistic perspectives without the constraints of rigid protocol adherence or formal quality appraisal. Although narrative synthesis may introduce selection bias and no formal risk-of-bias assessment was performed, deliberate efforts were made to minimize this limitation by applying a structured literature search and study selection process and by incorporating representative and influential studies published throughout the predefined search period (2008–2026), thereby providing a balanced and comprehensive overview of the current understanding.

## 3. Results

### 3.1. Microbiota Development

The very beginning of microbiota development is yet to be defined, while for many years the theory of “the sterile womb” has been widely accepted, the more recent literature showed the possibility of in utero colonization [8]; however, the major bacterial colonization occurs during birth when the newborn is exposed to the microorganisms of the birth canal, rectum and skin of the mother [9]. Due to the different exposures, there are some differences between the babies born via vaginal canal or cesarean section; when born by vaginal delivery, the infant gut is colonized by anaerobic strains such as *Escherichia coli*, *Staphylococcus* and *Streptococcus*, which create the optimal environment for the development of *Bacteroides* and *Bifisobacterium* species. In infants born by cesarean section, there is an early colonization with bacteria that are located on the mother’s skin, while other species such as *Bacteroides* and *Bifisobacterium* are found later; moreover, high concentrations of *Clostridium difficile* were observed [4]. Significant differences are shown until 3–6 months of life, after which they are substantially reduced. Delivery by cesarean section has been associated with an increased risk of neonatal respiratory distress syndrome, asthma, celiac disease, type 1 diabetes and obesity; research shows that the difference in microbiota might be a cause [4]. The type of feeding has also been investigated as a crucial determinant in the development of the microbiome.

Approximately 40% of the infant gut microbiota comes from maternal sources, particularly human milk and areolar skin, underscoring the critical role of breastfeeding in the initial microbial colonization and development of the infant gut microbiota [10].

Infants who have been breastfed show higher counts of genera thought to be beneficial such as *Bifidobacterium*, *Lactobacillus*, and *Clostridia* [10]; breastfeeding plays such a beneficial role in the development of the infant microbiota that it is shown that the microbiota of exclusively breastfed infants born via c-section is very similar to that of those born by vaginal delivery [11]. When breastfeeding is not feasible, formula supplemented with prebiotics or probiotics can shift the microbiota of formula-fed infants closer to that of breastfed infants [12]. Even in the early stages of life, it is possible to notice how the microbiota plays a crucial role in the development of future illnesses; therefore, this should be taken into account when counseling parents regarding their newborn’s diet.

### 3.2. Eubiosis and Diet

A balanced microbiota might be the key to a long and heathy life. The term eubiosis refers to the normal, healthy state of balance among microorganisms (such as bacteria, fungi, and yeasts) living within the human body, particularly in environments like the gut, skin, or mouth. In this balanced state, beneficial microbes thrive and support the host’s overall health, while harmful pathogens are kept in check.

The gut microbiome plays a crucial role in maintaining health as it is involved in many human biological processes via several mechanisms such as nutrient extraction, metabolism and immunity. The gut microbiota is equipped with unique enzymes [13] that human cells lack, which are used for the biosynthesis of bioactive molecules such as vitamins, essentials amino acids and folates and for bile acid metabolism [14].

Diet is one of the most important factors affecting the intestinal microbiome; changes in both food intake quality and quantity can cause changes in microbial composition [15]. Each dietary component has a direct effect on the microbiota and therefore on the host.

The Mediterranean diet has been proven to sustain a healthier microbiota by increasing Bacteroidetes and certain Clostridium groups, while decreasing Proteobacteria and Bacillaceae phyla [16]. Extra virgin olive oil enhances lactic acid bacteria and their bioactive products, correlating with reductions in pro-inflammatory mediators such as IL-6, IL-17A, TNF-α, IL-1β, COX-2, LDL-c, and oxidized LDL-c [17]. It is known that seafood is rich in omega-3 fatty acids, which favor the growth of *Lachnospiraceae* and *Bifidobacteria* families, while decreasing LPS-producing Enterobacteria and thus having positive effects on anti-inflammatory properties [18]. The Mediterranean diet is also rich in dietary fibers, vitamins and minerals, while the consumption of red meat is limited; all of these have been shown to promote a diversified microbiota and immune system, which is associated with a healthier life. On the other hand, excessive consumption of red meat promotes changes in microbiota composition with increasing concentrations of *Fusobacterium nucleatum*, *Streptococcus bovis*/*gallolyticus*, *Escherichia coli*, and *Bacteroides fragilis*, which can cause hyperproliferation of colon enterocytes and alteration of the gut barrier, eventually leading to gastrointestinal complications and even colorectal cancer [19].

### 3.3. Short-Chain Fatty Acids

One crucial role of the gut microbiota is the production of short-chain fatty acids that are used as local and systemic signaling molecules [20]. Short-chain fatty acids (SCFAs) are organic acids whose carbon chain is composed of less than six carbon atoms. Among these, acetate, propionate and butyrate are the most represented and studied [21]. SCFAs are involved in preserving the integrity and permeability of the gut barrier by upregulating the genes involved in the synthesis of proteins that make up tight junctions and in the strengthening of the mucus layer [22]; as explained later in the paper, the integrity of the intestinal barrier is a key factor in many of the pathological pathways in which the microbiota is involved. SCFAs are also involved in the differentiation and apoptosis of enterocytes [23]. SCFA receptors are located on the surface of many different cells such as pancreatic cells, neurons, and neutrophiles [24]. The most important SCFA is butyrate; therefore, the bacteria that produce this are essential for maintaining a healthy gut environment. The main species involved in butyrate production are the *Lachnospiraceae* and *Ruminococcaceae* families [25]; the production of this SCFA requires oxygen consumption, which creates an anaerobic environment that is hostile to some pathogenic bacteria such as *Salmonella* and *Escherichia coli* [26].

Short-chain fatty acid (SCFA) levels change throughout life, mirroring alterations in the composition of the microbiome [27].

In the early stages of life, the composition of the gut microbiota evolves quickly. Initially, *Enterobacteriaceae* is the most abundant; then, *Bifidobacteriaceae* and, at the end of breastfeeding, Firmicutes reach a concentration that is similar to that in an adult [28]. Changes in the composition of the microbiota reflect on the production of SCFAs at different stages of life. In infancy, acetate is found in higher concentrations because it is the main byproduct of Bifidobacteria, which are predominant in the early gut microbiota. In later stages of childhood, the composition of the microbiota shifts once more, with an increased prevalence of *Enterobacteriaceae*. During adulthood, a more varied diet causes other changes in the composition of the intestinal microbiome. Finally, in the elderly, a decrease in microbial diversity and an increase in Proteobacteria is seen [29]. 

### 3.4. Dysbiosis and Chronic Inflammation

The proper functioning of the human gut microbiota is strictly related to a well-balanced microbial composition, predominantly comprising members of the Bacteroidetes, Firmicutes, and Actinobacteria phyla, with Proteobacteria present in lower proportions. Any change in the composition of the microbiota, such as disruptions in the relative abundance of these taxa or the emergence of atypical microbial groups, can result in a state of microbial imbalance, called dysbiosis. Dysbiosis is commonly associated with a reduction in microbial diversity and an overrepresentation of Proteobacteria. An expanding body of evidence suggests that this altered microbial state is linked to a range of pathological conditions and may contribute to both the onset and progression of various diseases [30].

The immune system plays a crucial role in sustaining a balanced relationship with the gut microbiota by promoting a non-inflammatory state of homeostasis and vice versa. This tolerance is mediated through several coordinated mechanisms. A key component is the mucus barrier, which acts as a physical shield to reduce direct microbial contact with the intestinal epithelium. In addition, the host secretes antimicrobial peptides and immunoglobulin A (IgA), both of which contribute to controlling microbial populations without provoking an inflammatory response. These strategies allow the immune system to distinguish between beneficial commensals, which are preserved, and potential pathogens, thereby preserving intestinal health and preventing inappropriate immune activation. Disruption of this balance can contribute to dysbiosis and is implicated in the pathogenesis of various inflammatory and autoimmune disorders [31].

Altered gut microbiota can elevate levels of interleukin-18 (IL-18), which suppresses mucin production by interfering with the maturation of goblet cells [31]. IL-18 also downregulates IL-22-binding proteins, which are involved in tissue repair and the regulation of antimicrobial peptide expression [32]. When intestinal barrier permeability is compromised, there is an enhanced translocation of bacteria across the gut epithelium. Under normal conditions, these bacteria are cleared by T helper 1 (TH1) and T helper 17 (TH17) lymphocytes, whose activation is strongly promoted by polysaccharides derived from *Bacteroides* spp. [33]. Alterations in the gut microbiota also significantly impair the maturation of the innate immune system, as commensal microorganisms are key modulators of its development. In germ-free or dysbiotic conditions, neutrophils and dendritic cells (DCs) exhibit diminished functional capacity, characterized by reduced microbial killing and decreased secretion of type I interferons (IFN-Is) and interleukin-15 (IL-15), respectively [34].

### 3.5. Inflammaging

**Figure 1:**
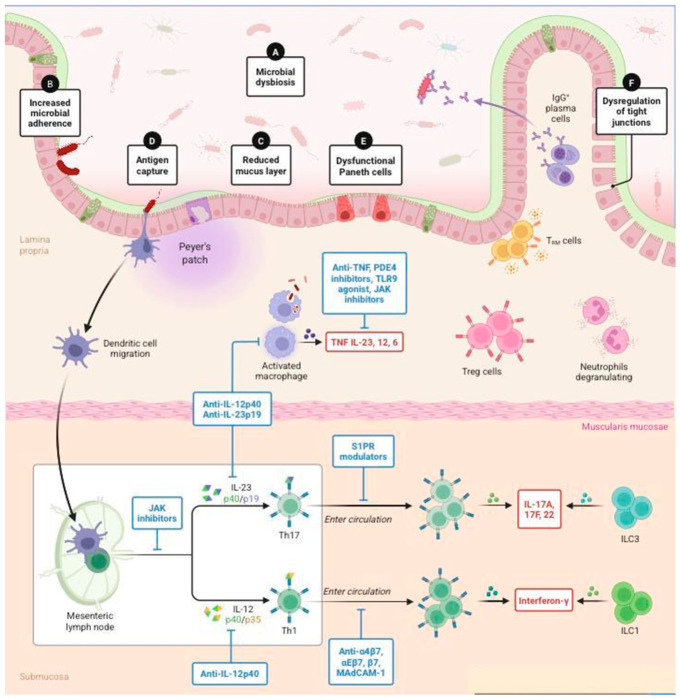
Dysbiotic gut and inflammatory pathway.

**Figure 2:**
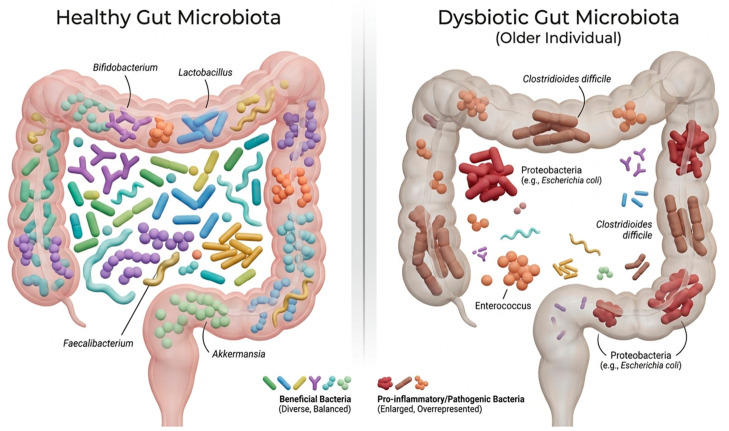
Comparison between healthy gut microbiota and dysbiotic gut microbiota.

Advancing age is closely associated with a gradual decline in immune system regulation, with one of the most prominent manifestations being chronically elevated levels of pro-inflammatory signals in the circulation. This low-grade, systemic inflammation—referred to as “inflammaging”—is marked by increased concentrations of some pro-inflammatory molecules, called cytokines such as IL-1, IL-6, IL-8, IL-13, and IL-18, as well as IL-1 receptor antagonist (IL-1RN), C-reactive protein (CRP), serum amyloid A, type I interferons (IFN-α and IFN-β), transforming growth factor-beta (TGF-β), and tumor necrosis factor (TNF) along with its soluble receptors [35]. Emerging evidence suggests that age-related alterations in the gut microbiota contribute significantly to this inflammatory milieu. In people who have reached 100 years or more, it is possible to detect high levels of anti-inflammatory molecules; this fine equilibrium between pro- and anti-inflammatory molecules is probably what allows them to approach the biological limit of longevity [36]. Dysbiosis in the elderly is characterized by reduced microbial diversity and a decline in beneficial commensal species, which can compromise epithelial barrier integrity and promote systemic immune activation through increased microbial translocation and stimulation of pro-inflammatory pathways (Figure 1). Changes in the gut microbiota also favor the systemic inflammation process; these include a progressive decline in some species of bacteria such as the ones responsible for butyrate production, such as *Faecalibacterium* and *Roseburia*, known for their anti-inflammatory effects; a reduction in microbial diversity; and an increased relative abundance of potentially pathogenic, low-abundance taxa or pathobionts, such as members of the families *Enterobacteriaceae*, *Streptococcaceae*, and *Staphylococcaceae* [37]. The immune system and the gut microbiota influence each other creating a loop such that it is not possible to define what comes first; however, as the global population grows older, it is crucial to understand these mechanisms (Figure 2).

## 4. Discussion

### 4.1. Oral–Gut–Brain Axis

The gut and the brain engage in bidirectional communication via neural pathways and signaling molecules capable of crossing the blood–brain barrier. A key component of this interaction is the vagus nerve, which directly connects enteric neurons with the central nervous system [38]. The gut microbiome produces substances that reach the central nervous system through the vascular and lymphatic systems; neurons also interact with the gut microbiome via numerous neurotransmitters [39]. Another pathway seems to involve the microbial production and release of various neuroactive compounds, including short-chain fatty acids (SCFAs) and biogenic amines such as serotonin, dopamine, and histamine, as well as amino acid-derived metabolites like gamma-aminobutyric acid (GABA) and tryptophan derivatives. These substances may act as neurotransmitters or neuromodulators, thereby affecting both gut and brain function [39]. An additional pathway through which the gut microbiota may affect brain function is the production of neurotoxic metabolites. Some gut microbes are capable of synthesizing harmful compounds, including D-lactic acid and ammonia, which, under certain conditions, can enter the bloodstream and exert negative effects on the central nervous system [40]. Alterations in the gut microbiota have been linked to a range of neurological disorders, including mood-related conditions such as anxiety and depression, as well as more-severe pathologies like neurodegenerative diseases and drug-resistant forms of epilepsy [41]. Blood analyses in individuals with brain amyloidosis and cognitive decline have shown elevated levels of pro-inflammatory cytokines, accompanied by an increased abundance of pro-inflammatory gut bacteria, such as *Escherichia*/*Shigella*, and a reduction in anti-inflammatory species like *Eubacterium rectale* [42]. Brain changes linked to dysbiosis that may contribute to the development of Alzheimer’s disease seem to arise through several overlapping mechanisms. As mentioned earlier, gut microbes can affect the levels of key neurotransmitters. Beyond this, research suggests that the microbiome may also influence proteins and receptors that are important for synaptic plasticity, such as NMDA receptors, brain-derived neurotrophic factor (BDNF), serotonin receptors, and serotonin itself. Inflammation is another major factor in this process. When dysbiosis occurs, it can lead to a state of chronic neuroinflammation, with increased production of pro-inflammatory cytokines and a less balanced immune response. Under normal conditions, the gut microbiota also helps support the production of neuroprotective compounds, so when this balance is disrupted, the brain may become more vulnerable to AD [43].

Clinical and experimental evidence supporting a connection between gut microbiota and Alzheimer’s disease (AD) has led to the development of the “age-related dysbiosis” hypothesis. This theory suggests that AD may emerge as a consequence of immune system aging. As individuals age, significant shifts occur in the gut microbiota composition because of immune system dysregulation. Notably, there is an increase in potentially harmful groups of bacteria, such as *Proteobacteria*, while beneficial probiotics like *Bifidobacteria* decline. Additionally, the production of neuroprotective molecules, such as short-chain fatty acids (SCFAs), decreases [44]. These changes in microbiota composition are thought to contribute to neuroinflammation, a key feature in the pathogenesis of AD. Moreover, studies have shown that the loss of microbiome function, particularly affecting genes involved in the production of SCFAs, correlates with elevated levels of pro-inflammatory cytokines in healthy elderly individuals. These factors together may possibly enhance susceptibility to neurodegenerative diseases, including AD [45]. Gut microbial composition is also affected by periodontal pathogens via enteral transmission or indirectly via hematogenous transmission [46].

In recent years, the role of the oral microbiome in neurodegenerative diseases has gained increasing attention as epidemiological evidence supports an association between oral health deterioration, neuropathology, and cognitive decline [47]. The biological plausibility of this oral–brain axis is supported by evidence showing that periodontal pathogens and their products can promote systemic inflammation, disrupt blood–brain barrier integrity, and contribute to neuroinflammatory processes, thereby influencing central nervous system homeostasis [48]. Adnan et al. demonstrated significant differences in oral microbiota according to cognitive performance, with individuals showing lower cognitive function exhibiting higher abundances of pro-inflammatory taxa, including *Treponema*, *Parvimonas*, and *Filifactor* [49]. It is now well established that extracellular vesicles (EVs) mediate intercellular communication within the central nervous system and participate in bidirectional signaling between the brain and peripheral tissues [50]. Accordingly, Mulet et al. identified oral microbiome-derived proteins in brain-derived EVs from the post-mortems of patients with Alzheimer’s disease and vascular dementia, further demonstrating that oral microbiome-derived EVs can cross the blood–brain barrier and interact with proteins involved in amyloid-β and tau pathology, providing a potential mechanistic link between oral dysbiosis and neurodegeneration [51]. Oral health has also been found to play an important role in mental health; as was found in a large cohort study by Malan-Müller et al., the composition of the overall oral microbiome was significantly impacted by several mental health variables [52].

### 4.2. Cardiovascular Diseases

The gut microbiome appears to play a significant role in modulating inflammation associated with cardiovascular disease (CVD). Metagenomic profiling of fecal samples has revealed that individuals with unstable atherosclerotic plaques exhibit notable alterations in microbial composition compared with those with stable plaques. In particular, a reduction in some genera such as *Roseburia*, which are known to exert anti-inflammatory effects, has been observed. Moreover, the microbial community in these patients shows an enhanced potential to produce pro-inflammatory molecules, such as peptidoglycans, alongside a diminished capacity to synthesize protective, anti-inflammatory compounds like carotenes. These microbial imbalances may contribute to the chronic inflammatory state that underlies plaque instability, thereby implicating the gut microbiota as a key player in the progression and severity of CVD [53]. In individuals with high blood pressure, studies have identified a state of microbial dysbiosis marked by reduced diversity and a shift in the balance between beneficial and potentially harmful bacterial taxa. Notably, reductions in butyrate-producing bacteria, such as *Odoribacter*, have been linked to elevated blood pressure, while their presence correlates with improved vascular function [54]. One of the main pathways through which the gut microbiota may influence blood pressure regulation is via the production of SCFAs. These microbial metabolites are known to impact host physiology by interacting with G-protein-coupled receptors (GPRs), which are involved in processes such as renin release and vascular tone regulation [55]. SCFAs also exert anti-inflammatory effects and help maintain the integrity of the intestinal barrier, indirectly affecting systemic blood pressure.

Additionally, there is evidence suggesting that microbial metabolites can influence the autonomic nervous system, potentially modulating sympathetic activity, which is closely linked to hypertension [56].

Although gut microbiota alterations have been consistently associated with cardiovascular diseases, their causal role remains uncertain. The current evidence suggests that dysbiosis may contribute to disease progression through inflammatory and metabolic pathways, but it may also result from the disease itself, lifestyle changes, or pharmacological treatments.

### 4.3. Diabetes Mellitus Type 2

Dysbiosis has been observed in patients diagnosed with diabetes mellitus, though the microbial alterations differ between type 1 diabetes (T1DM) and type 2 diabetes (T2DM). In T1DM, which is characterized by autoimmune destruction of insulin-producing β-cells, dysbiosis typically manifests as an increase in *Bacteroidetes* and *Clostridium*, along with a decrease in beneficial genera such as *Bifidobacterium*, *Lactobacillus*, and *Prevotella*, many of which are known for their mucin-degrading and anti-inflammatory properties [57]. In T2DM, particularly when not associated with obesity, dysbiosis is characterized by a decline in *Clostridium* and a reduced abundance of Bacteroidetes, other than an increase in Lactobacillus. Both forms of diabetes are commonly associated with decreased microbial diversity, especially reduced populations of SCFA-producing bacteria, and impaired intestinal barrier integrity. This can result in increased gut permeability, allowing detrimental bacterial components such as lipopolysaccharides (LPSs) to translocate into the circulation and therefore be responsible for systemic complications. This condition, called metabolic endotoxemia, drives systemic inflammation and promotes insulin resistance, which is a defining feature of T2DM. Although it is not yet fully clear whether dysbiosis is a trigger or a consequence of diabetes, several longitudinal and interventional studies suggest that imbalances in the gut microbiota can appear before the onset of disease, particularly in T1DM. For instance, in non-obese diabetic (NOD) mouse models, those raised in germ-free environments develop diabetes at higher rates than those housed in specific pathogen-free (SPF) conditions, implying that the presence of specific commensal bacteria may offer protective effects against autoimmune responses [58]; however, a direct correlation is yet to be found in human beings. The association between gut dysbiosis and type 2 diabetes appears to be bidirectional.

### 4.4. Osteoporosis

Recent studies have shown that gut microbes can also be a key regulatory factor in bone homeostasis [59]. The gut microbiota has emerged as a central regulator of bone health through multiple interconnected pathways that influence both mineral metabolism and bone remodeling. Certain microbial strains, notably *Lactobacillus* and *Bifidobacterium*, enhance the intestinal absorption of vital electrolytes such as calcium, magnesium, and phosphorus by modifying gut pH and optimizing the solubility and bioavailability of these nutrients. This contributes directly to improving bone mineral density (BMD), which is critical for skeletal strength. Moreover, the microbiota supports the synthesis of essential micronutrients like vitamin K and various B vitamins, which play indispensable roles in bone matrix formation and osteoblast function. Another key mechanism involves the microbial fermentation of dietary fibers into short-chain fatty acids (SCFAs), particularly butyrate and propionate. These metabolites exert anti-inflammatory effects and directly influence bone metabolism by inhibiting osteoclast differentiation and activity, thereby reducing bone resorption while preserving bone formation. SCFAs also promote the metabolic reprogramming of osteoclasts, reducing their capacity to degrade bone tissue. Furthermore, an imbalanced or dysbiotic microbiota can contribute to systemic inflammation, which negatively impacts bone turnover and may accelerate bone loss [60]. Emerging evidence suggests that targeting the gut microbiota—through probiotics, prebiotics, or dietary interventions—may offer a novel therapeutic strategy for preventing or mitigating conditions such as osteoporosis, especially in aging populations in which bone fractures can be fatal and where both gut microbial diversity and bone mineral density tend to decline [61].

Although growing evidence supports a role for the gut microbiota in bone metabolism, most available data are derived from preclinical or observational studies. Whether microbial alterations represent a causal factor, a disease modifier, or a consequence of aging and lifestyle changes remains unclear, highlighting the need for further clinical research.

While the gut microbiota has been the most extensively studied in the context of aging and disease, emerging research highlights that other human microbial niches—including the oral cavity, lungs, skin, vagina, and genito-urinary tract—also undergo significant changes with age and may play a role in the development of age-related diseases. Although studies in these areas are still relatively limited, the current evidence suggests that microbiota alterations in these sites might be associated with increased susceptibility to common conditions in older adults, such as pneumonia, chronic obstructive pulmonary disease (COPD), urinary tract infections, reactive airway diseases, and various malignancies. These findings underscore the importance of considering the broader human microbiome beyond the gut in aging research and clinical practice [62].

## 5. Limitation

This review has several limitations. It was conducted as a narrative synthesis rather than a systematic review, precluding formal quality assessment and quantitative comparison of studies. The literature search was primarily limited to PubMed, potentially overlooking relevant research indexed in other databases. Additionally, the narrative approach may introduce selection and publication biases. The conceptual variability in defining the microbiota, alongside its evolving understanding and current knowledge gaps, further complicates interpretation. These factors should be considered when evaluating the findings and highlight the need for more-rigorous systematic analyses in future research.

## 6. Conclusions

The maintenance of a balanced and diverse microbiota through the use of probiotics, prebiotics, and targeted dietary interventions has gained increasing attention as a potential strategy to mitigate inflammaging, the chronic, low-grade systemic inflammation associated with aging [63]. A growing body of evidence indicates that age-related alterations in the gut microbiota—characterized by reduced microbial diversity and a shift toward pro-inflammatory taxa—play a critical role in the dysregulation of immune function and the pathogenesis of age-associated diseases [64].

Probiotics are live microorganisms that, when consumed in adequate amounts, can provide beneficial effects to health. They have been associated with improvements in the integrity of the intestinal epithelial barrier, as well as with the regulation of both innate and adaptive immune functions, ultimately contributing to a reduction in systemic inflammatory status. In a similar way, prebiotics consist of non-digestible food components that are selectively utilized by beneficial gut bacteria, stimulating their growth and activity. This process enhances the production of short-chain fatty acids (SCFAs), including butyrate, that play a key role in modulating inflammation, acting both at the level of the gut and in more-distant tissues throughout the body [65]. Moreover, dietary patterns rich in fiber, polyphenols, and unsaturated fats have been positively correlated with microbiota diversity and the maintenance of mucosal immune homeostasis, whereas Western-style diets, contrary to the Mediterranean diet, are high in saturated fats and refined sugars and are associated with dysbiosis and elevated pro-inflammatory markers (Table 1).

Future research should focus on well-designed longitudinal cohort studies to better define the temporal relationship between microbiota alterations and disease onset, thereby helping to distinguish causal mechanisms from secondary microbial changes associated with disease progression. In parallel, adequately powered randomized controlled trials are required to evaluate the clinical efficacy and long-term safety of microbiota-targeted interventions, including probiotics, prebiotics, synbiotics, dietary modulation, and other emerging therapeutic strategies. Furthermore, the integration of multi-omics technologies—such as metagenomics, metatranscriptomics, metaproteomics, metabolomics, and extracellular vesicle profiling—offers a promising opportunity to achieve a more comprehensive understanding of host–microbiome interactions and to identify novel biomarkers and therapeutic targets. Finally, future investigations should extend beyond the gut microbiota to explore the role of other microbial ecosystems, particularly the oral microbiome and the oral–gut–brain axis, which are increasingly recognized as important contributors to systemic inflammation, neurodegeneration, and the pathophysiology of age-related diseases. Such advances will be essential for the development of personalized microbiome-based preventive and therapeutic strategies.

In conclusion, strategies aimed at preserving or restoring microbiota equilibrium via probiotic and prebiotic supplementation, alongside dietary modulation, represent a promising adjunct in the prevention and attenuation of inflammaging.

Further mechanistic studies and well-designed clinical trials are needed to elucidate the precise interactions between the gut microbiota and host inflammatory pathways and to determine the optimal formulations and regimens for microbiome-targeted interventions in aging populations as the use of such interventions is growing at a fast pace.

## Figures and Tables

**Table 1:** Schematic overview of the review.

Section	Main Topic	Key Concepts
Microbiome development	Establishment of the microbiota throughout life	Birth mode, breastfeeding, early microbial colonization, immune maturation
Eubiosis and diet	Factors maintaining a healthy microbiota	Mediterranean diet, dietary fiber, microbial diversity, beneficial metabolites
Short-chain fatty acids (SCFAs)	Functional role of microbial metabolites	Butyrate, acetate, propionate, intestinal barrier, immune regulation
Dysbiosis and chronic inflammation	Consequences of microbial imbalance	Reduced diversity, increased intestinal permeability, immune dysregulation
Inflammaging	Aging-associated immune dysfunction	Chronic inflammation, microbiota changes, immunosenescence
Gut–brain axis	Communication between microbiota and CNS	Neural, endocrine and immune pathways, neuroinflammation
Cardiovascular diseases	Microbiota and vascular health	SCFAs, inflammation, blood pressure regulation
Type 2 diabetes mellitus	Microbiota and glucose metabolism	Gut permeability, endotoxemia, insulin resistance
Osteoporosis	Microbiota and bone metabolism	Calcium absorption, vitamin synthesis, SCFAs
Therapeutic perspectives	Microbiota modulation	Probiotics, prebiotics, dietary interventions

## Data Availability

No new data were created or analyzed in this study. Data sharing is not applicable to this article.
